# Untangling the interaction of α-synuclein with DNA i-motifs and hairpins by volume-sensitive single-molecule FRET spectroscopy†

**DOI:** 10.1039/d1cb00108f

**Published:** 2021-07-02

**Authors:** Sanjib K. Mukherjee, Jim-Marcel Knop, Rosario Oliva, Simone Möbitz, Roland Winter

**Affiliations:** Physical Chemistry I-Biophysical Chemistry, Department of Chemistry and Chemical Biology, TU Dortmund University, Otto-Hahn Strasse 4a Dortmund D-44227 Germany roland.winter@tu-dortmund.de

## Abstract

The intrinsically disordered protein α-synuclein causes Parkinson's disease by forming toxic oligomeric aggregates inside neurons. Single-molecule FRET experiments revealed conformational changes of noncanonical DNA structures, such as i-motifs and hairpins, in the presence of α-synuclein. Volumetric analyses revealed differences in binding mode, which is also affected by cellular osmolytes.

## Abstract 2

The conformational landscape of noncanonical DNA structures is markedly affected by monomeric and aggregated α-synuclein, by osmolytes such as TMAO, and by pressure.

## Graphical abstract

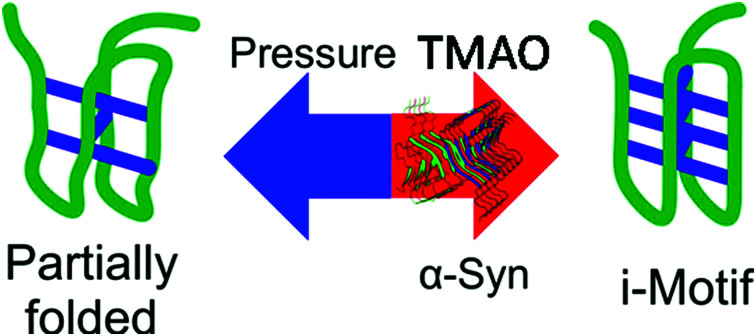

α-Synuclein (α-Syn) is a small intrinsically disordered presynaptic protein which regulates neurotransmitter vesicle cycling, but is, under pathological conditions, also closely associated with Parkinson's disease (PD). PD arises because of abnormal aggregation of α-Syn, and these aggregates are dominantly found in Lewy bodies as the hallmark of PD.^1–3^ The human α-Syn protein has 140 amino acid residues and consists of three distinct regions, which include an amphipathic N-terminal domain (residues 1-60), a central hydrophobic region (the non-Aβ component (NAC) region with residues 61-95), and a highly negatively charged proline-rich C-terminal domain (residues 96-140). The conformation of the polypeptide has been found to be markedly affected in the presence of lipid membranes and cosolutes.^4–7^ The toxic aggregated form of α-Syn detected in PD affected brain as well as other amyloidogenic proteins, such as the prion protein (PrP), have a high propensity to interact also with DNA.^3,8–12^ Therefore, studying the conformation of DNAs in the presence of α-Syn has immense importance because the interaction with α-Syn forms can significantly affect DNA replication and transcription along with causing DNA damage.

Nucleic acids can fold into different noncanonical structures besides the well-known double helix, which are particularly prone to interact with amyloidogenic peptides. Noncanonical DNAs have been shown to play important roles in replication, transcription and translation.^13–18^ DNA hairpins (DNA-Hp, Fig. 1A) regulate gene expression, act as target sites for protein recognition and nucleation sites for higher order RNA structures.^19,20^ On the other hand, cytosine-rich (C-rich) sequences that form intercalated structures, known as i-motifs, which are formed by a neutral and protonated cytosine (C–C^+^) that bind through three H-bonds (Fig. 1B), are often seen in connection with cancer and tumor formation due to their role in promoter regions of oncogenes.^17,21^ Both, i-motif and G-quadruplexes (G4Qs) are also found in the telomeric region of chromosomes.^16^ The i-motif is folded at low pH of 5.0, while at neutral pH either random coil or partially folded structures are found. Interestingly, macromolecular crowding has been found to foster formation of the folded structure of the i-motif.^16^

**Fig. 1:**
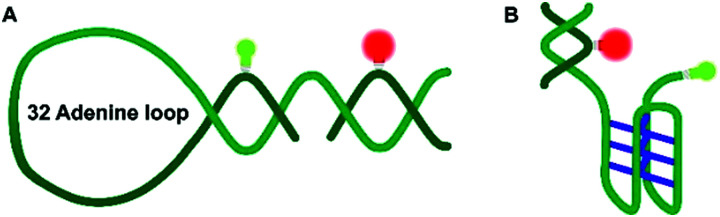
Schematic representation of the DNA Hp (A) and the hTel i-motif construct (B). The C–C^+^ H-bonds are shown in blue. The green and red bulbs represent the donor dye ATTO 550 and the acceptor dye ATTO 647N used in the smFRET experiments.

Many intracellular assemblies and processes rely on weak, often also transient interactions, sometimes collectively termed quinary interactions.^22,23^ They are generally highly susceptible to variations in their environment and respond sensitively to changes in temperature, pressure, pH, and the local concentration of surrounding cosolutes. Perturbation of the volume of the system, *e.g.* by applying osmotic or hydrostatic pressure, is particularly suited to detect and measure such weak interactions.^24–28^ Volume changes are generally a very mild perturbation that happen, for example, during cell-cycle changes or in response to deleterious pressure conditions, such as in the deep sea where organisms have to cope with pressures up to the 1 kbar range.^29^ Volume modulation can also be employed to study the binding affinity and stoichiometry of weakly bound complexes.^24,30^ As the conformational properties of intrinsically disordered peptides, such as of α-Syn, are strongly influenced by the solution and environmental conditions, such approach represents a valuable strategy to explore also the free-energy landscape and disclose rare conformational substates of biomolecules.^24–28^ In fact, it has been found that α-Syn, insulin, lysozyme and PrP aggregates are affected by high hydrostatic pressure (HHP).^24,31–38^ NMR data by Roche *et al.*^31^ suggested a slight increase in the population of polyproline II regions and a slight decrease of transient α-helical conformations of α-Syn at about 2 kbar. Interestingly, Silva, Foguel and coworkers showed that α-Syn fibrillar structures, by perturbing the cavity-rich hydrophobic core of the fibrils and pushing water inside, dissociate by pressures in the 1–2 kbar pressure range.^32,33^ In addition, pressure-dependent studies have shown that they can provide novel information about the polymorphic states that fibrillar aggregates can adopt.^24,32–38^ Moreover, pressure-axis experiments have also been carried out to explore the conformational landscape of DNA hairpins, G-quadruplexes and i-motifs, and it has been found that, different from the rather pressure-stable B-DNA, that noncanonical DNA and RNA structures are more susceptible to pressure modulation.^18,24,39–44^

To gain a better molecular-level and mechanistic understanding of the interaction of α-Syn in its monomeric and aggregated state with such noncanonical chromosomal DNA sequences, we carried out conformation-sensitive single-molecule Förster resonance energy transfer (sm-FRET) experiments in concert with the pressure perturbation approach. Pressure-dependent confocal sm-FRET experiments to explore folding reactions and conformational transitions of nucleic acids have been successfully introduced, recently.^42–44^ This method avoids ensemble averaging, which enables us to elucidate the conformational dynamics of the noncanonical DNA structures and how they are affected by the interaction with monomeric and aggregated α-Syn also in a pressure-dependent manner, allowing us to extract volumetric changes accompanying the structural conversions. As paradigmatic examples, we utilized a DNA hairpin (DNA Hp) and a telomeric i-motif (hTel i-motif) of sequence (CCCAAT)_3_CCC with a double-strand part for the second FRET label (for details, see the ESI†). To mimic potential cellular osmolyte effects, measurements have also been carried out in the presence of trimethylamine-*N*-oxide (TMAO), one of the most potent organic cellular osmolytes, which is also found in deep-sea organisms having to cope with extreme temperatures and pressures.^23,29,45,46^

The peaks in the FRET efficiency histograms are related to conformations with different spatial separations, *R*, of the two attached dyes and thus different FRET efficiencies, *E*, as *E* = *R*^6^_0_·(*R*^6^_0_ + *R*^6^)^−1^. The Förster radius, *R*_0_, is the distance at which 50% of the excited donor molecules will be deactivated; here, *R*_0_ = 6.5 nm for the fluorophores used, Atto 550 and Atto 647N. The FRET efficiency distribution of the i-motif in buffer solution at pH 7.4 shows a single peak centered at *E* ≈ 0.65 (Fig. 2A), which represents a partially folded conformation of the i-motif, while in acidic pH 5.0, this peak shifts towards a higher efficiency of *E* ≈ 0.90, representing the fully folded conformation. Upon pressure application up to 1.5 kbar, no changes are observed in the *E*-histograms, *i.e.*, no conformational change (*e.g.*, complete unfolding) is observed (Fig. 2A and B), indicating dense packing and a lack of cavities in the partially folded state of the i-motif. In the presence of 1 M TMAO, a similar observation was made (Fig. ESI† S1A and B).

**Fig. 2:**
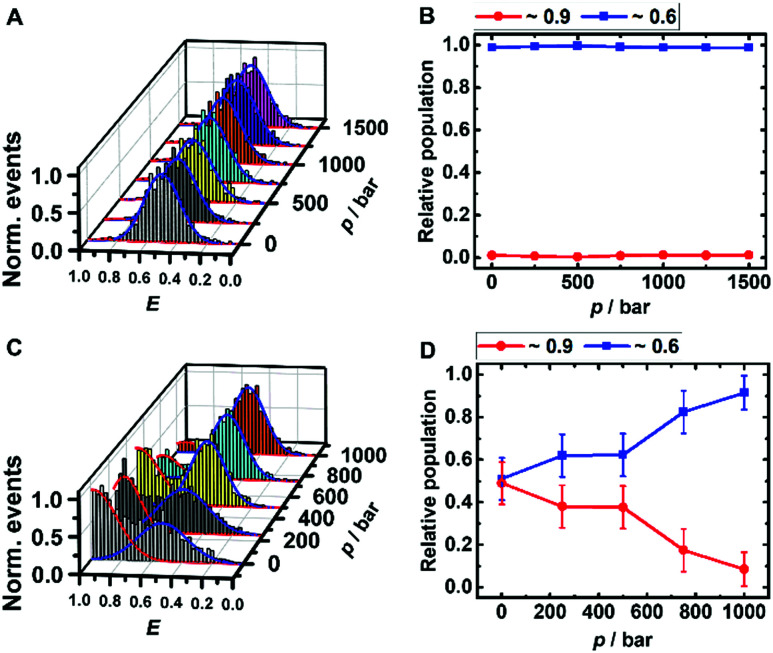
Pressure dependent smFRET-histogram (A and C) and population distribution (B and D) of the folded (red) and partially folded (blue) conformation of the i-motif (∼50 pM) in 20 mM Tris HCl, pH 7.5, at 25 °C. The samples for C and D contained additional 100 μM α-Syn oligomers.

Remarkably, a conformational transition of the i-motif was observed in the presence of both monomeric (Fig. ESI† S1C and D) and oligomeric (Fig. 2C and D) α-Syn, *i.e.* the interaction with the polypeptide promotes complete folding of the i-motif which is otherwise seen in acid pH, only. In the FRET histograms, two separate peaks emerged, with efficiencies *E* ≈ 0.9 (25%) and *E* ≈ 0.6 (75%) upon addition of the monomeric α-Syn, and with *E* ≈ 0.9 (50%) and *E* ≈ 0.6 (50%) after adding oligomeric α-Syn. In the presence of 1 M TMAO and monomeric α-Syn, the fraction of folded conformations of the i-motif increases further, from 25% (peptide only) to about 40% (peptide + TMAO). This can be rationalized by the chaperone activity of TMAO, which promotes compact conformational states^23,45^ and acts synergistically in stabilizing the folded structure of the i-motif. Both conformations of the i-motif remain essentially unaffected by pressure, pointing to the formation of a compact, void-free and hence pressure-stable i-motif-α-Syn complex (Fig. ESI† S1E and F). Complementary circular dichroism (CD) spectroscopic measurements confirmed the absence of a conformational change of monomeric α-Syn in the presence of TMAO (Fig. ESI† S5A). Differently, a drastic pressure effect on the conformation of the i-motif was observed in the presence of aggregated α-Syn. The fraction of the folded i-motif conformation decreased significantly (from 50% to 10%) with a concomitant increase of the partially folded conformation of the i-motif (Fig. 2C and D). We may assume that HHP dissociates the aggregates, leading to monomeric α-Syn with increasing pressure amplitude due to a weakening of hydrophobic interactions, loosening of intermolecular packing, and rupture of intermolecular salt bridges resulting in electrostriction.^23,24^ As HHP favors lower volume states, destabilization of the α-Syn aggregates toward states with less void volume and packing defects can be expected.^32–34^ This scenario would also be in agreement with own ThT fluorescence spectroscopic measurements on aggregated α-Syn solutions, revealing pressure-induced dissociation of the aggregated α-Syn species (Fig. ESI† S5B). From the pressure dependence of the population distribution, a volume change of Δ*V =* −54 cm^3^ mol^−1^ could be determined. Of note, similar Δ*V* values were found for the dissociation of lysozme (Δ*V* = −52.7 cm^3^ mol^−1^) and α-Syn (Δ*V* = −65 cm^3^ mol^−1^) amyloid.^34,47^ In the presence of the cosolute TMAO, a stabilization of the folded conformation (from 50% to 80%) was observed.

The DNA Hp is about 60% folded (*E* ≈ 0.9) and 40% unfolded (*E* ≈ 0.3) at ambient conditions, in good agreement with literature data (Fig. 3A and B).^42^ The population of the unfolded state increases up to 60% upon pressurization to 1 kbar, which corresponds to a volume change upon unfolding of the DNA Hp of Δ*V =* −26 cm^3^ mol^−1^ (Table 1 summarizes all volumetric data). TMAO leads to an increase of its pressure stability (Fig. ESI† S3), which is reflected in a smaller volume change of Δ*V* = −12 cm^3^ mol^−1^, and, again, is most likely due to the preferential exclusion of the TMAO from the hydrated surface of the hairpin's backbone. Similarly, in the presence of monomeric α-Syn, the population of folded conformers of the DNA Hp increases by ∼10% (Fig. 3D and ESI† Fig. S4A), and the pressure stability of the folded conformer has increased as well (Δ*V* = −18 cm^3^ mol^−1^), it is less than in 1 M TMAO (Δ*V* = −12 cm^3^ mol^−1^), however (Fig. ESI† S3C and D). Similar to the i-motif, the population of the folded conformer increases also in the presence of aggregated α-Syn, and increasing pressure leads to a destabilization of the folded state (Δ*V* = −12 cm^3^ mol^−1^). No such pressure-induced destabilization was observed in the presence of both aggregated α-Syn and 1 M TMAO (Δ*V* ≈ 0 cm^3^ mol^−1^).

**Fig. 3:**
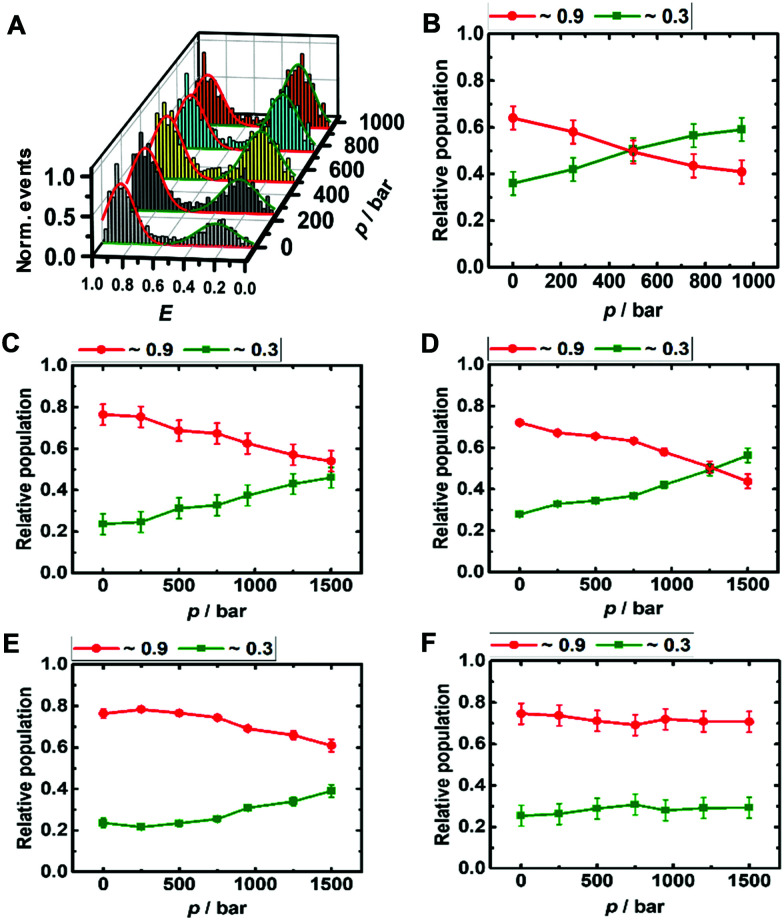
Pressure dependent smFRET *E*-histogram and population distribution of the open (*E* ≈0.9) and closed (*E* ≈ 0.3) conformation of the DNA Hp (∼50 pM) in 20 mM Tris HCl, pH 7.5, and 15 mM NaCl at 25 °C (A and B). The samples further contained (C) 100 μM α-Syn monomers, (D) 100 μM α-Syn monomers + 1 M TMAO, (E) 100 μM α-Syn aggregates, and (F) 100 μM α-Syn aggregates + 1 M TMAO.

**Table 1:** Pressure-induced volume change of the conformational transitions of the hTel i-motif and DNA Hp under different solution conditions. Δ*V* was calculated from the pressure dependence of the equilibrium constant, dln *K*/d*p =* −Δ*V*/(*RT*), of the relative population distributions *via* linear regression

Sample	Δ*V*/cm^3^ mol^−1^
hTel i-motif in 100 μM α-Syn aggregate	−54 ± 10
DNA Hp in buffer	−26 ± 2
DNA Hp in 1 M TMAO	−12 ± 3
DNA Hp in 100 μM monomeric α-Syn	−18 ± 1
DNA Hp in 100 μM monomeric α-Syn + 1 M TMAO	−19 ± 3
DNA Hp in 100 μM aggregated α-Syn	−14 ± 2
DNA Hp in 100 μM aggregated α-Syn + 1 M TMAO	∼0

Altogether, significant effects on the conformational landscape of the noncanonical DNA structures were observed upon addition of monomeric and aggregated α-Syn, and compatible osmolytes such as TMAO impose an additional effect on the population distribution of the conformational states of the i-motif and DNA Hp. Marked effects have previously also been observed for G4Qs.^46^ Although the mode of interaction between α-Syn and the different noncanonical DNA structures looks similar, the specificity and the strength of such interactions depend on the internal architecture and solvent-exposed surface of the DNA structures. Of note, several earlier studies also found that DNA (*via* electrostatic and hydrophobic interactions, showing also some specificity for GC nucleotide sequences in its binding ability), osmolytes such as TMAO, glycerol, betaine, and taurine as well as crowding agents can have significant effects on the conformational landscape of α-Syn, which might alter its affinity towards other biomolecules and its aggregation and fibrillation propensity.^48–52^ Generally, DNA–protein interactions depend on electrostatic and non-specific interactions between the amino acid residues and the DNA backbone and its bases. Interactions with α-Syn can be envisioned to take place *via* its positively charged N-terminus and the phosphate backbone of the DNA, and *via* hydrogen bonds and hydrophobic interactions of amino acid residues and the DNA bases. As the i-motif is comprised of a cytosine-rich sequence and the DNA Hp contains 32 largely exposed adenine residues in the loop region, differences of the interaction strength with α-Syn can be expected. α-Syn has 17 glutamate residues along with 3 asparagines and 6 glutamines. Earlier reports suggested that glutamine and asparagine amino acid residues form a large number of hydrogen bonds with adenine bases, and glutamate is responsible for a maximum number of hydrogen bonds with cytosine,^53^ which might help stabilize the folded state of the i-motif in the presence of α-Syn. Further stabilization is observed by the osmolyte TMAO owing to its excluded volume effect, which is similar to what has been observed in the presence of crowding agents, which tend to stabilize the more compact folded state as well.^46^ Based on the same mechanism, TMAO also suppresses pressure-induced dissociation of the oligomeric α-Syn. The volumetric data reveal that different from the oligomeric case, where pressure leads to dissociation of α-Syn aggregates, the monomeric α-Syn-i-motif complex is densely packed and pressure stable. Formation of a cavity-rich aggregated α-Syn-i-motif complex can be deduced from the volume change of −54 cm^3^ mol^−1^ upon pressure perturbation. Pressure-insensitivity of the monomeric α-Syn-i-motif complex suggests binding largely though H-bonds, which are known to be even strengthened at HHP.^24^ Different from the pressure-stable partially folded state of the i-motif, the folded state of the DNA Hp is rather pressure sensitive, indicting less dense packing of the bases, *i.e.* the existence of void volume in the folded state. Both, monomeric and oligomeric α-Syn lead to a significant stabilization of the folded state of the DNA Hp. Additional stabilization of the folded state is achieved by the excluded volume effect imposed by TMAO, which renders the polypeptide-DNA Hp complex cavity-free and pressure stable up to at least 1 kbar.

To conclude, depending on the surrounding cellular components, the intrinsically disordered α-Syn molecule can adopt various conformations and several aggregation states.^54,55^ As its aggregates are responsible for different neurological disorders, their interaction with noncanonical DNA structures as studied here might help reveal hidden mechanisms of the associated diseases. Further, high hydrostatic pressure can significantly impact noncanonical DNA inside living cells and thus affect genetic profiles in deep-sea organisms. Hence, next to the potential to help detect conformational substates and classify weak interactions, pressure studies on DNA–protein interactions can provide additional mechanistic information to understand the physico-chemical properties of these biomolecules under such harsh environmental conditions.^29,56^

## Conflicts of interest

The authors declare no competing financial interests.

## Supplementary Material

CB-002-D1CB00108F-s001
