# Supplementary material for: Untangling the interaction of α-synuclein with DNA i-motifs and hairpins by volume-sensitive single-molecule FRET spectroscopy
Source: RSC Chem Biol. 2021 Jul 2;2(4):1196–200. doi: 10.1039/d1cb00108f (PMC8341996; doi:10.1039/d1cb00108f)
Supplement: CB-002-D1CB00108F-s001 [file CB-002-D1CB00108F-s001.pdf]

Electronic Supplementary Material (ESI) for RSC Chemical Biology.
This journal is © The Royal Society of Chemistry 2021

Supporting Information

Table of Contents

S.1 Experimental and Methods Section

S.2 Supporting References

S.3 Supporting Figures

S.1 Experimental and Methods Section

Materials and sample preparation

TMAO and Thioflavin-T were purchased from Sigma-Aldrich (Germany) and used as received without further purification. As described by Baltierra-Jasso et al.,[1] the DNA oligonucleotides were synthesized and fluorescently labeled by IBA life solutions GmbH (Goettingen, Germany). The sequences of these oligonucleotide strands are:

C1: 5'-TGG GGA CGG CGG A CCC TAA CCC TAA CCC TAA CCC Y-3',
C2: 5'-CCG XCG CCG TCC CCA-3'
H2:5'TGGCGACGGCAGCGAGGCTTAGCGGCAAAAAAAAAAAAAAAAAAAAAAAAAAAAA AA AGC CGC X-3',
A2: 5'-GCC TCG CYG CCG TCG CCA-3',

(X is T-Atto 550 and Y is T-Atto 647N).

Annealing of the two oligonucleotide strand pairs (C1 & C2, H2 &A2) were carried out in a buffer containing 20 mM Tris-HCl, 50 mM NaCl at pH 7.5. For annealing, the elongated strand (H2 or C1) was mixed with the complementary strand (A2 or C2) in a molar ratio of 1:1 (both at 1 µM after mixing) and annealed by first heating at 95 °C for 5 min, followed by gradually cooling down to room temperature at a rate of -0.5 °C/min using a thermocycler. The buffer solution used in these measurements contains 20 mM Tris-HCl (pH 7.4) and was filtered by a 0.45 µm sterile Whatman Puradisc 30 syringe filter. After dilution, smFRET measurement were carried out at a DNA concentration of ~50 pM, and the NaCl concentration was ~2.5 µM for the measurements in buffer and with α-Syn.

Expression and purification of α-Synuclein

The expression and purification of α-synuclein was carried out as described before.[2,3] The plasmid was purchased from GenScript. The plasmid pT7-7 expressing human α-synuclein was transformed into *Escherichia coli* strain BL21 (DE3). A single colony was picked and inoculated into 100 mL LB medium containing 150 µg/mL ampicillin and grown at 37 °C with shaking at 250 rpm until the absorbance at 600 nm reached 0.8. Induction was then carried out by adding 1 mM IPTG (final concentration) and the culture was further grown under similar conditions for 3 h. The cells were harvested, resuspended in 0.75 mL of buffer (50 mM Tris-HCl, pH 7.5, 10 mM EDTA and 150 mM NaCl) and frozen at -80 °C. Tubes containing frozen cells were placed in a boiling water bath for 7 min and the supernatant collected after centrifugation at maximum speed for min. Streptomycin sulfate (136 µL/mL of supernatant) and glacial acetic acid (228 µL/mL of supernatant) were added and centrifuged for 2 min. Again, the supernatant was recovered and precipitated with ammonium sulfate (saturated ammonium sulfate at 4°C was used 1:1, v/v, with supernatant). The protein was collected as a precipitate by centrifugation and washed once with 1 mL of ammonium sulfate solution (4 °C, 1:1, v/v, saturated ammonium sulfate and water). The washed pellet was resuspended in 900 µL of 100 mM ammonium acetate (to form a cloudy solution) and

precipitated by adding an equal volume of ethanol at room temperature. Precipitation with ethanol was repeated once more. The pellet was resuspended in 100 mM ammonium acetate and extensively dialyzed against 10 mM Tris-HCl buffer, pH 7.4.

Preparation of α-synuclein aggregates

Lyophilized α-Syn was dissolved in 20 mM Tris-HCl buffer (pH 7.4) in the presence of 10% ethanol and 100 mM NaCl at 37 °C without stirring for 6 h, followed by incubation at room temperature to perform all the measurements.

Single-molecule FRET (smFRET) measurements

SmFRET measurements were carried using a confocal fluorescence microscope (MicroTime 200, PicoQuant) under freely diffusing conditions. The pulsed interleaved excitation (PIE) FRET technique was used to separate dually labeled from singly labeled species.[4] Briefly, in the PIE FRET technique, both the donor and acceptor are alternatively excited by the laser pulse. First, a laser pulse of suitable wavelength excites the donor and then another laser pulse excites the acceptor independently from FRET after a certain time delay (50 ns, 20 MHz repetition rate), allowing us to calculate the photon stoichiometry, *S*, which is the ratio of photons emitted after donor excitation and the sum of total photons emitted after donor and direct acceptor excitation. For a donor-only species, $S = 1$, and for an acceptor-species only, $S = 0$. A green laser pulse at 560 nm (LDH series, PicoQuant) and a red laser pulse at 635 nm (LDH series, PicoQuant) are used to excite the donor Atto 550 and acceptor Atto 647 N, respectively. A quad band dichroic mirror (ZT 405/488/561/640, Chroma) was used to reflect both the green and red laser light to the entrance port of the fluorescence microscope. Donor and acceptor fluorescence signals were separated to two different detection channels, first by using a dichroic mirror (FF 650 Di01, Semrock), followed by band pass filters FF 01-593/40 (Semrock) and FF 01-676/29 (Semrock). Two SPCM-AQR series single photon avalanche diodes (SPAD) were used as detection channels for the donor and acceptor fluorescence.

The peaks in the FRET efficiency histograms are related to conformations with different spatial separations, R, of the two attached dyes and thus different FRET efficiencies, E, as $E = R_0^6 \cdot \left(R_0^6 + R^6\right)^{-1}$. The Förster radius, R_0, is the distance at which 50% of the excited donor molecules will be deactivated. Here, $R_0 = 6.5$ nm for the fluorophores used, Atto 550 and Atto 647N.[5,6]

Fluorescence Spectroscopy

The high-pressure steady state fluorescence was recorded by using a K2 fluorometer from ISS, Inc. (Champaign, IL, USA) at the temperature of 25 °C. The instrument is equipped with a xenon arc lamp as light source. Thioflavin-T (ThT), a commonly used probe to monitor *in vitro* amyloid fibril formation, was used as an extrinsic fluorophore for the ensemble measurements of the dissociation process of α-synuclein-aggregates as a function of pressure. Upon binding to amyloid fibrils and cross-β-aggregates, ThT gives a strong fluorescence signal at approximately 485 nm. ThT was used in a molar ratio of 2:1 to α-synuclein. The excitation and emission wavelengths were set at 430 nm and 485 nm, respectively.

CD measurements

Far-UV circular dichroism spectra were acquired on a Jasco J-715 (Jasco Corportation, Tokyo, Japan). Far-UV spectra of protein samples were recorded in the range 260-195 nm using a 0.01 cm path length quartz cuvette. The spectra recorded are the results of 6 accumulations.

References

1. L. E. Baltierra-Jasso, M. J. Morten, L. Laflör, S. D. Quinn and S. W. Magennis, *J. Am. Chem. Soc.* 2015, **137**, 16020–16023.
2. M. J. Volles and P. T. Lansbury, *J. Mol. Biol.* 2007, **366**, 1510-1522.

3. R. Shaltiel-Karyo, M. Frenkel-Pinter, N. Egoz-Matia, A. Frydman-Marom, D. E. Shalev, D. Segal and E. Gazit, *PLoS One*, 2010, **5**, e13863.
4. S. Ruettinger, V. Buschmann, B. Kraemer, S. Orthaus and F. Koberling, *Appl. note, PicoQuant GmbH*, 2013.
5. L. Arns and R. Winter, *Chem. Comm.* 2019, **55**, 10673-10676.
6. S. Patra, C. Anders, N. Erwin and R. Winter, *Angew. Chemie. Int. Ed.* 2017, **56**, 5045-5049.

Supporting Figures

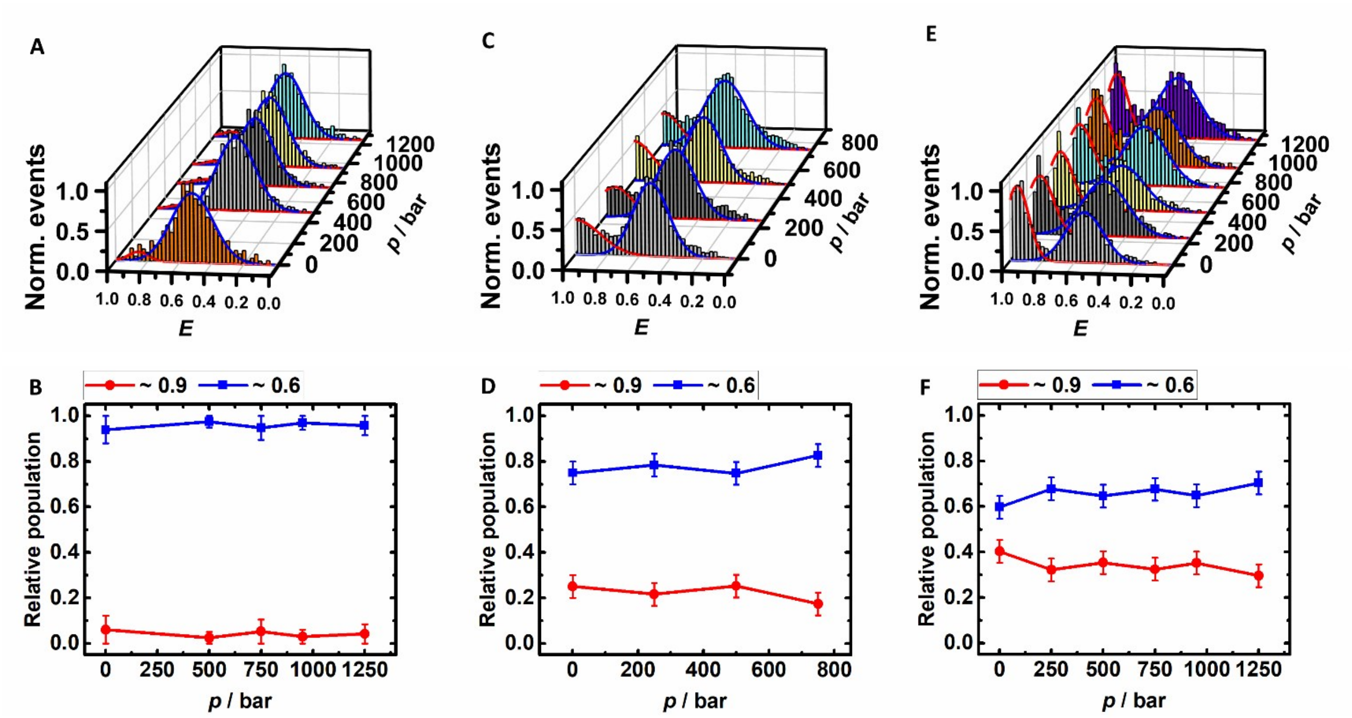

Figure SI 1: Pressure dependent smFRET-histogram (A, C, E) and relative population distribution (B, D, F) of the hTel i-motif sequence (~50 pM) in 20 mM TrisHCl, pH 7.5, at 25 °C. The samples additionally contained 1 M TMAO (A, B), 100 µM α-Syn monomers (C, D) and 1 M TMAO + 100 µM α-Syn monomers (E, F).

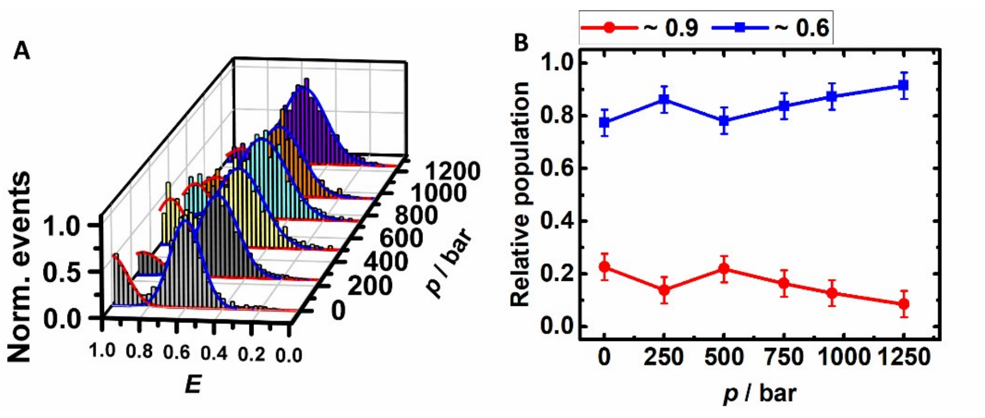

Figure SI 2: Pressure dependent smFRET-histogram (A) and population distribution (B) of the hTel i-motif sequence (~50 pM) in 20 mM Tris HCl, pH 7.5, 100 µM α-Syn aggregates and 1 M TMAO at 25 °C.

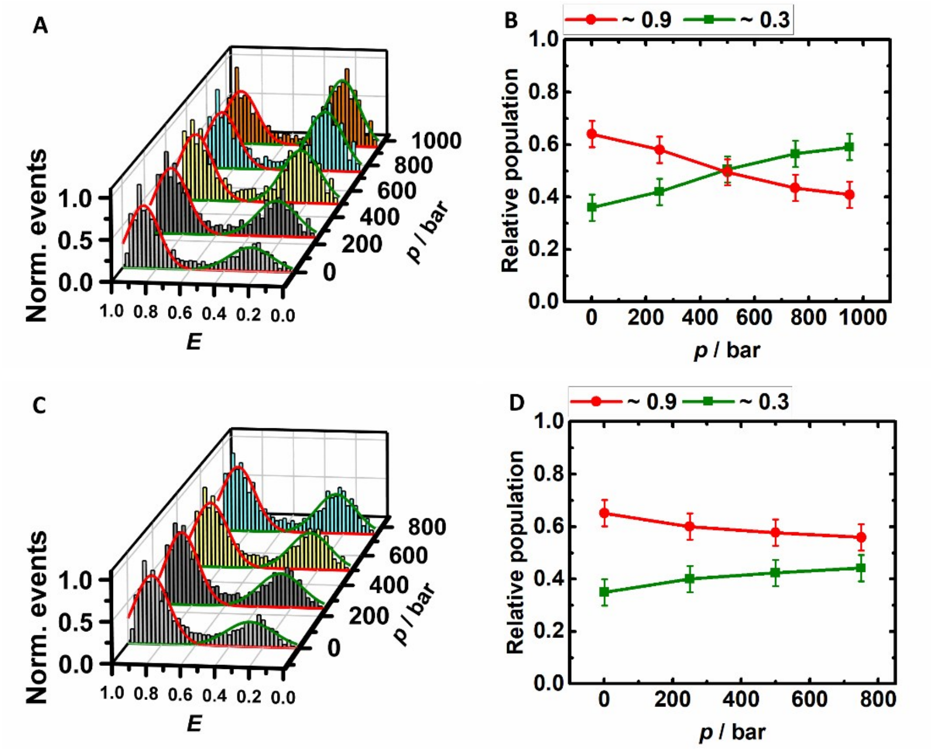

Figure SI 3: Pressure dependent smFRET histogram (A, C) and population distribution (B, D) of DNA HP (~50 pM) in 20 mM TrisHCl and 15 mM NaCl at 25 °C. The samples for C and D contained additional 1 M TMAO.

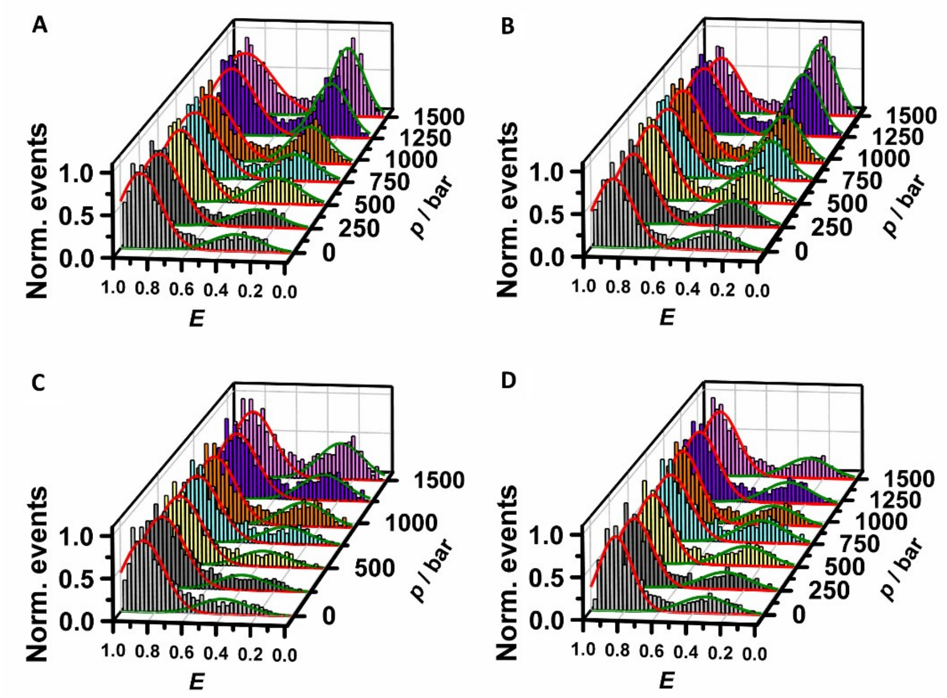

Figure SI 4: Pressure dependent smFRET histograms of DNA Hp (~50 pM) in 20 mM TrisHCl pH 7.5 and 15 mM NaCl at 25 °C. The samples further contained (A) 100 μM α-Syn monomers, (B) 100 μM α-Syn monomers + 1 M TMAO, (C) 100 μM α-Syn aggregates and (D) 100 μM α-Syn aggregates + 1 M TMAO.

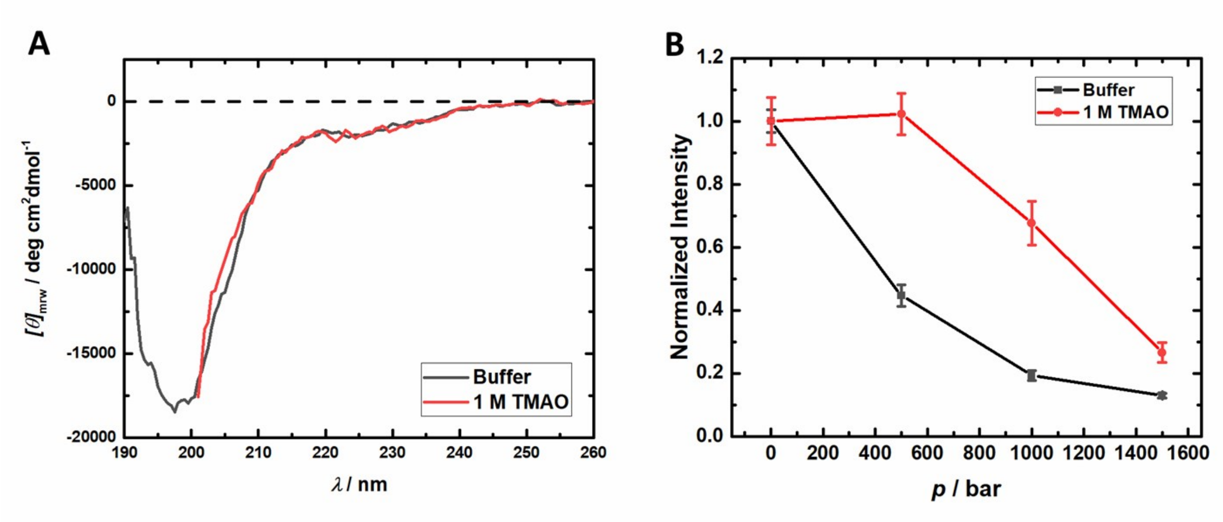

Figure SI 5: (A) CD-measurement of monomeric α-Syn in buffer and 1 M TMAO. (B) Pressure dependent ThT fluorescence intensity of aggregated α-Syn (oligomers) in pure buffer and buffer+ 1 M TMAO.
